# Global research trends and hotspots of exosome-mediated drug delivery across the blood-brain barrier: a bibliometric study from 2015 to 2025

**DOI:** 10.3389/fphar.2026.1835883

**Published:** 2026-06-10

**Authors:** Zhipeng Chen, Yang Jiang, Xingxiao Yin, Yanqi Li, Hezhi Sai, Yanping Song, Zhen Shen, Qigang Chen

**Affiliations:** 1 School of Physical Education, Yunnan Normal University, Kunming, China; 2 Department of Rehabilitation, Kunming Municipal Hospital of Traditional Chinese Medicine, The Third Affiliated Hospital of Yunnan University of Chinese Medicine, Kunming, China

**Keywords:** bibliometrics, blood-brain barrier, central nervous system diseases, drug delivery systems, exosomes, extracellular vesicles

## Abstract

**Background:**

The blood-brain barrier (BBB) is a major obstacle to targeted drug delivery for central nervous system (CNS) diseases. Although liposomes and polymeric nanoparticles have improved brain drug delivery, limitations remain in BBB targeting, long-term biocompatibility, and *in vivo* clearance. Exosomes are endogenous nanoscale extracellular vesicles with favourable biocompatibility, low immunogenicity, and BBB-crossing potential. Therefore, this bibliometric study summarises the current research status, future research trends, and challenges in the more specific field of exosome-mediated BBB drug delivery.

**Methods:**

A comprehensive search was conducted across the Web of Science Core Collection (WoSCC), PubMed, and Embase databases for relevant English-language literature on exosome-mediated drug delivery across the blood-brain barrier from 2015 to 2025. WoSCC served as the primary source for bibliometric analysis. PubMed and Embase databases were used for supplementary validation. Software such as VOSviewer, CiteSpace, and R-bibliometrix was employed for literature visualisation analysis.

**Results:**

This study included 1,365 relevant articles from the WoSCC database, and the annual publication volume showed a steady upward trend. China and the United States significantly lead in both the number of publications and the number of core contributing institutions in this field. Co-occurrence analysis of keywords showed that research hotspots are mainly focused on exosomes, the blood-brain barrier, drug delivery, and Alzheimer’s disease. PubMed and Embase were used as supplementary validation databases, including 1,089 and 1,517 records, respectively. Their annual publication trends, major countries/regions, core journals, and keywords/themes were generally consistent with WoSCC, supporting the macro-level stability of the bibliometric findings.

**Conclusion:**

Unlike previous bibliometric analyses that mainly focused on overall trends in CNS exosome research, this study focuses specifically on the direction of exosome-mediated drug delivery across the BBB. The findings show a shift from basic vesicle characterisation toward engineered delivery systems, CNS disease applications, and translational evaluation. Mammalian-derived exosomes remain dominant, while plant-derived vesicles, AI-assisted design, biomimetic hybrid nanovesicles, and gut–brain axis strategies are emerging areas of focus. Future research should prioritise systematic platform comparisons, standardised evaluation, quality control, scalable production, long-term safety, and regulatory pathways.

## 1 Introduction

With the acceleration of global population ageing, central nervous system (CNS) diseases, represented by Alzheimer’s disease (AD), Parkinson’s disease (PD) and brain tumours, have become major factors contributing to high disability and mortality rates worldwide (Dong, 2018). Epidemiological data indicate that over the past 30 years, the absolute number of deaths caused by CNS diseases has increased by 39%, and disability-adjusted life years (DALYs) have risen by 15%, with this trend being particularly evident in low- and middle-income countries (Feigin et al., 2020). However, existing treatments still face significant challenges, among which the physiological barrier of the blood-brain barrier (BBB) serves as a core bottleneck that limits the effective delivery of drugs to brain lesions and hinders precise treatment of CNS diseases (Zhang et al., 2025; Yang, 2025; Abbott, 2013). The BBB is a semi-permeable barrier between the brain and the peripheral circulatory system, and, as a core component of the neurovascular unit (NVU), it maintains homeostasis within the brain microenvironment through selective permeability mechanisms while blocking exogenous toxins, pathogens, and abnormal immune cells from entering the central nervous system (Chaulagain et al., 2023; Banks, 2016; Ayloo and Gu, 2019).

This interception function is primarily manifested physiologically as a synergistic effect between physical and biochemical barriers. The physical barrier is composed of brain microvascular endothelial cells, the basal membrane, pericytes, and astrocytes (Pandit et al., 2020). Among them, the tight junctions between endothelial cells form the core of the physical barrier, directly restricting the free diffusion of polar molecules and macromolecules and almost completely blocking the paracellular pathway for hydrophilic substances (Greene and Campbell, 2016; Bauer et al., 2014). The biochemical barrier mainly refers to the highly expressed ABC transporter protein family on the endothelial cell membrane, among which the most typical are P-glycoprotein (P-gp) and breast cancer resistance protein (BCRP) (Tournier and Langer, 2025; Chai et al., 2022). These transporter proteins utilise energy provided by ATP to actively expel lipophilic substances that have already crossed the cell membrane into endothelial cells back into the blood against the concentration gradient (Nicolazzo and Banks, 2011). The combined effect of this physical barrier and biochemical efflux results in extremely low drug entry efficiency into the brain parenchyma. Research data indicate that nearly 100% of macromolecular drugs (such as monoclonal antibodies, recombinant proteins, and gene therapies) and over 98% of small-molecule drugs cannot cross the BBB to achieve effective therapeutic concentrations (Pandit et al., 2020; Banks, 2016). This extremely low drug delivery efficiency has become the main bottleneck currently restricting CNS drug development.

In order to circumvent or physically open the BBB, researchers have attempted methods such as hyperosmotic mannitol, focused ultrasound, increasing drug lipophilicity or increasing the dosage; however, these strategies are often accompanied by risks such as neurotoxicity, cerebral oedema, non-targeted distribution and systemic toxicity (Han, 2021; Wu et al., 2023; Nájera-Maldonado et al., 2025). Therefore, nanomedicine delivery systems have gradually become an important direction in CNS drug delivery research. Synthetic nanocarriers such as liposomes and polymer nanoparticles offer advantages in improving drug stability and enhancing drug loading capacity, but still face challenges with BBB penetration efficiency, biocompatibility, and *in vivo* clearance (Ekhator et al., 2023). In comparison, exosomes, which are endogenous extracellular vesicles with diameters of 30–150 nm and a lipid bilayer structure derived from host cells, exhibit extremely low immunogenicity and greater circulatory stability (Ha et al., 2016; Mehdizadeh et al., 2025). Furthermore, exosomes can cross the BBB via receptor-mediated transcytosis and accumulate in damaged brain tissue or neuronal regions (Alvarez-Erviti et al., 2011; Abdelsalam et al., 2023). Based on these characteristics, exosomes have received continuous attention as drug delivery carriers for CNS diseases.

Existing bibliometric studies have analysed the overall development trends of CNS exosome research, but these studies mostly focus on the general research landscape of exosomes in CNS diseases, with insufficient attention to the more specific drug-delivery scenario of “exosome-mediated crossing of the BBB”. Unlike previous studies, this study employs a bibliometric approach, not focusing on the annual updates of general CNS exosome research, but systematically analysing the literature related to exosome-mediated BBB-crossing drug delivery, outlining publication trends, collaboration networks, keyword evolution, and research hotspots, and further discussing future research directions and translational challenges in this field, thereby providing a more focused reference for the design of exosome-based BBB-targeting drug delivery systems and clinical translational research.

## 2 Materials and methods

### 2.1 Search strategy

This study primarily used the WoSCC database for retrieval. PubMed and Embase databases were introduced as external validation sets for clinical relevance. Comparative verification ensured the comprehensiveness and reliability of the retrieval results. Due to differences in field structure and indexing rules across the three databases, the literature retrieved from them was not merged after retrieval. Data extraction and analysis were conducted separately to clarify retrieval differences and ensure consistent research results.

Due to the significant increase in research on exosome engineering, nanodrug delivery systems, and BBB-targeted delivery after 2015, this period better reflects exosomes moving from basic research to brain-targeted drug delivery. Meanwhile, 2025, as the most recent year with complete data available, helps illustrate the changes in the knowledge structure in the more specific research direction of exosomes crossing the BBB over the past decade. Therefore, the search period was set from January 1, 2015, to December 31, 2025. The search strategy was based on MeSH terms and free-text terms used in related thematic studies, and was preliminarily constructed around the three conceptual groups of “exosomes/extracellular vesicles,” “blood-brain barrier,” and “delivery/penetration/transport/targeting” to ensure the search focused on BBB-related drug delivery research rather than generalised CNS exosome studies (Mehdizadeh et al., 2025; Pedder et al., 2025; Rehman et al., 2023; Das et al., 2019). The final search strategy was constructed based on Boolean logic: in WoSCC, TS =(“Exosome*” OR “Extracellular Vesicle*” OR “EVs” OR “Exosomal” OR “Microvesicle*”) AND (“Blood-Brain Barrier” OR “BBB” OR “Blood-brain-barrier”) AND (“Cross*” OR “Penetrat*” OR “Deliver*” OR “Transport*” OR “Transcytosis” OR “Targeting”) was used; in PubMed, a supplementary search was conducted using (“Exosomes” [Mesh]) AND (“Blood-Brain Barrier” [Mesh]); in Embase, the search strategy was based on three core concept groups and adjusted according to its Emtree vocabulary and field rules. All three databases were limited to English-language publications, and only Article and Review document types were retained, with retracted publications deleted. Given that this study is a bibliometric analysis, the aim is to depict the macro-knowledge structure and research trends in exosome-mediated drug delivery across the BBB, rather than to evaluate intervention effects or the quality of evidence in individual studies. Therefore, the search results were limited by publication date, language, and document type using the database’s built-in filtering functions, and retracted articles were excluded without manual secondary screening or risk-of-bias assessment. Ultimately, 1365, 1089, and 1517 articles were selected from WoSCC, PubMed, and Embase, respectively. Supplementary Figure S1 presents the database search and screening process using a PRISMA 2020-style flowchart (Page et al., 2021). All data used in this study were directly sourced from public literature databases; ethical committee approval was not required.

In addition, AI, machine learning, and other related terms were not included as primary search terms in this study to avoid excessively narrowing the scope of the literature. Given that AI-related content is an emerging topic in discussions of future trends, a targeted supplementary search was conducted using the following terms: “exosome” OR “extracellular vesicle”, “drug delivery”, “artificial intelligence” OR “machine learning” OR “deep learning” OR “nanomedicine”. The results are used solely to support the discussion in Section 4.4 and are not included in the main text bibliometric analysis.

### 2.2 Analysis methods

The articles in the WoSCC database were exported in the formats “Full Record and Cited References” and “Plain Text”, with the file name set to “download_XXX.txt”. In this study, we employed tools such as CiteSpace (version 6.1. R6), VOSviewer (version 1.6.18), and R-bibliometrix (version 4.2.3) for bibliometric analysis. Specifically, CiteSpace was used to perform dual-map overlay analysis, identify citation bursts and keyword bursts, and generate a keyword clustering timeline map, with the time slice set from January 2015 to December 2025. VOSviewer was primarily used for the following analyses: country and institution analyses, co-citation analyses of authors, journals, and documents, and keyword co-occurrence analyses. The R-bibliometrix package was used to present the top ten publishing countries, authors, and journals, and to analyse the evolution of thematic trends in articles retrieved from the WoSCC database. In addition, Microsoft Excel 2019 was used for quantitative analysis of publication output.

The literature in the PubMed database was exported in PubMed format for comparative verification with WoSCC results, focusing on annual publication trends, country/region distribution, institutions, authors, journals, and keyword analysis. The literature from the Embase database was exported in RIS format and was not included in the complete bibliometric analysis, but was used for the final multi-database robustness verification in the results section. This verification mainly compared the consistency between WoSCC and PubMed, and WoSCC and Embase in terms of annual publication trends, major countries/regions, keywords, and core journal sources. Annual publication trends were evaluated using Pearson correlation analysis. For countries/regions, keywords, and journal sources, this study extracted the Top 10 items from each database, computed the Jaccard similarity coefficient to assess item overlap, and used Spearman’s rank correlation to assess the consistency of rankings across common items.

## 3 Results

### 3.1 Overview of publications

**FIGURE 1:**
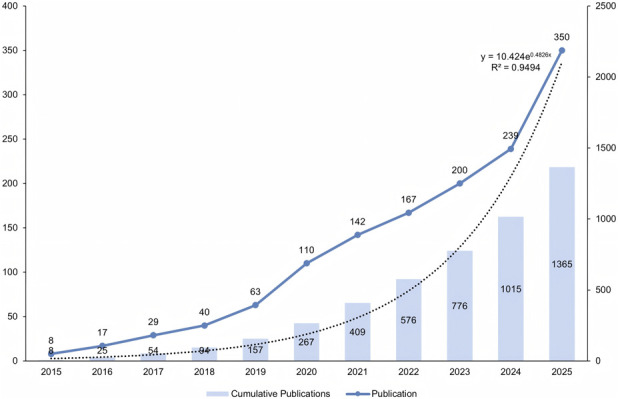
Annual publication volume and total cumulative publications (WoSCC).

**FIGURE 2:**
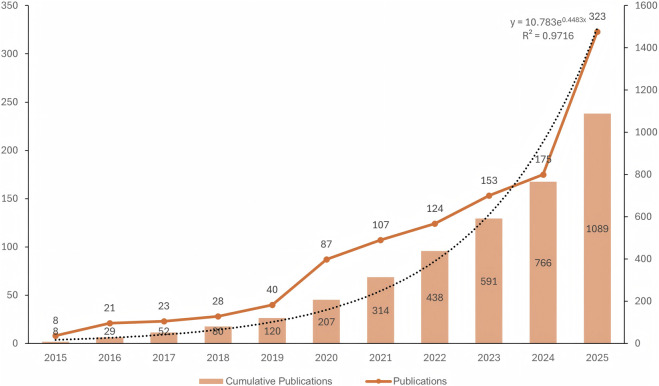
Annual publication volume and total cumulative publications (PubMed).

Initially, this study retrieved 1456 and 1161 articles from the WoSCC and PubMed databases, respectively. After systematic database screening, 1365 and 1089 articles were ultimately included for bibliometric analysis. Figures 1, 2 show that, between 2015 and 2025, the annual and cumulative publication volumes in both databases increased steadily, indicating that exosome-mediated drug delivery across the BBB is receiving increasing attention.

### 3.2 Country/region analysis

The distribution of publications by country/region is generally consistent between WoSCC and PubMed. Supplementary Figures S2, S3 show that in WoSCC, China ranks first with 577 publications, followed by the United States (218), India (72), Iran (55) and Italy (51); PubMed shows similar results, with China (379) still ranking first, followed by the United States (157), India (58), Iran (44) and South Korea (30).

**FIGURE 3:**
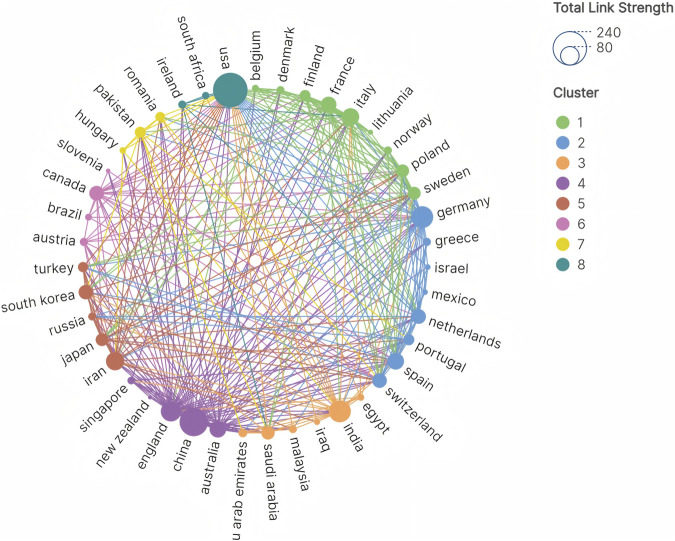
National collaboration network visualization map.

The national cooperation network further shows that international collaboration is mainly concentrated among high-output countries. Among them, the United States has the most collaborations with other countries, totalling 228, followed by China (155) and India (93), indicating that these countries play an important role in the global cooperation network (Figure 3).

### 3.3 Institutional analysis

There are certain differences among the high-output institutions in the two databases, which may be related to differences in database coverage and the differing methods of institutional indexing. In the WoSCC database, Harvard University has the highest number of publications, followed by the Chinese Academy of Sciences and Shanghai Jiao Tong University, most of which are comprehensive research entities; in the PubMed database, Nanjing University of Chinese Medicine ranks first, followed by the University of Coimbra and Central South University, most of which are medical schools and affiliated hospitals (Table 1 and 2).

**TABLE 1:** Top 10 institutions by publication volume in the WoSCC database.

Rank	Institution	Country	Article
1	HARVARD UNIVERSITY	USA	66
2	CHINESE ACADEMY OF SCIENCES	China	58
3	SHANGHAI JIAO TONG UNIVERSITY	China	54
4	TABRIZ UNIVERSITY OF MEDICAL SCIENCE	Iran	49
5	NANJING UNIVERSITY OF CHINESE MEDICINE	China	47
6	UNIVERSITY OF CALIFORNIA SYSTEM	USA	47
7	UNIVERSITY OF TEXAS SYSTEM	USA	46
8	ZHEJIANG UNIVERSITY	China	44
9	FUDAN UNIVERSITY	China	43
10	SICHUAN UNIVERSITY	China	42

**TABLE 2:** Top 10 institutions by publication volume in the PubMed database.

Rank	Institution	Country	Article
1	NANJING UNIVERSITY OF CHINESE MEDICINE	China	187
2	UNIVERSITY OF COIMBRA	Portugal	155
3	CENTRAL SOUTH UNIVERSITY	China	115
4	CAPITAL MEDICAL UNIVERSITY	China	90
5	SOUTHERN MEDICAL UNIVERSITY	China	81
6	TIANJIN MEDICAL UNIVERSITY GENERAL HOSPITAL	China	81
7	NANJING UNIVERSITY	China	65
8	SICHUAN UNIVERSITY	China	64
9	TABRIZ UNIVERSITY OF MEDICAL SCIENCES	Iran	62
10	SOUTHEAST UNIVERSITY	China	60

**FIGURE 4:**
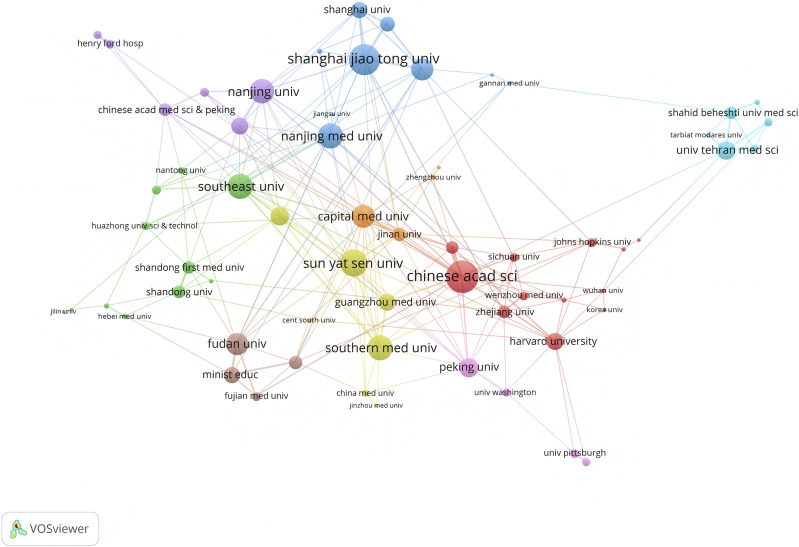
Institutional collaboration network visualization map (node size represents the total connection strength of the Institution, and the lines indicate the degree of collaboration/connection).

The institutional collaboration network shows that the Chinese Academy of Sciences is at the centre, with the highest connection strength (28), indicating its important bridging role in global collaboration. Harvard University has the highest cumulative citation frequency (2394), suggesting its strong academic influence in this field (Figure 4).

### 3.4 Author analysis

The volume of publications by authors shows consistency across the two databases, with Wang Y. and Zhang Y. both ranking among the most prolific authors (Supplementary Figures S4, S5).

**FIGURE 5:**
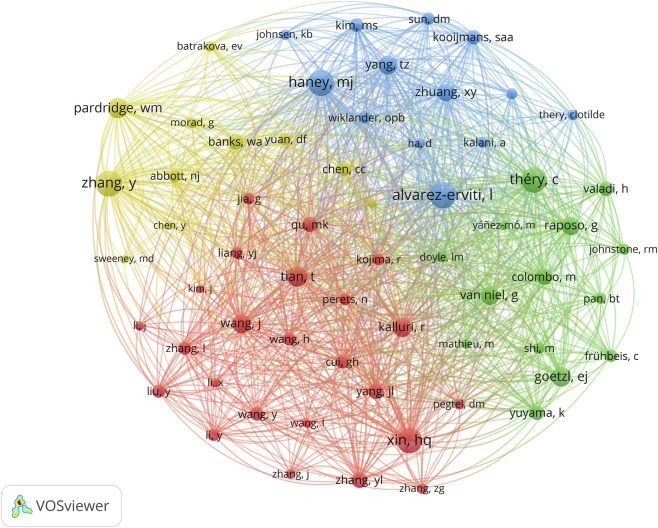
Network visualization map of author co-citation analysis (node size represents the total connection strength of the author, and the lines indicate the degree of collaboration/connection).

Co-citation analysis of authors indicates that Zhang Y (429 times), Alvarez-Erviti L. (424 times), and Théry C. (424 times) are the most frequently co-cited scholars, suggesting that their research has had a broad impact on the field of exosome-mediated BBB drug delivery (Figure 5).

### 3.5 Journal analysis

Research in this field is mainly published in journals in molecular biology, drug delivery, nanomedicine, and pharmaceutics. According to the WOSCC database, the *International Journal of Molecular Sciences* (72 articles, h-index 28, impact factor 4.9) has the highest number of publications, followed by the *Journal of Controlled Release* (48 articles, h-index 19, impact factor 11.5) and *Pharmaceutics* (44 articles, h-index 20, impact factor 5.5); the top three journals in PubMed are largely consistent with WoSCC, indicating that the main publication platforms are fairly stable across different databases (Supplementary Figures S6, S7).

**FIGURE 6:**
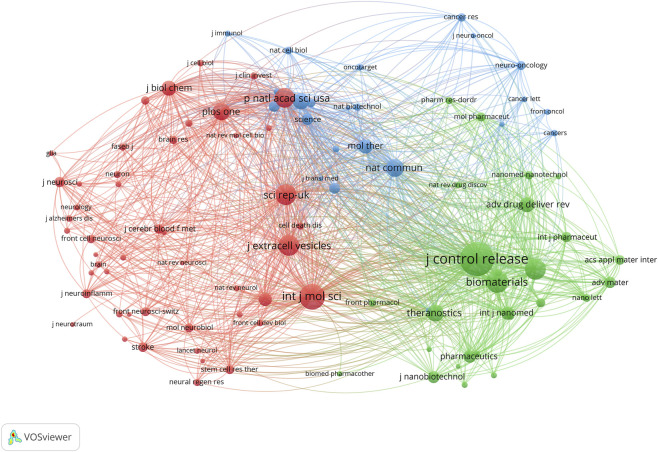
Network visualization map of journal co-citation analysis (node size represents the total connection strength of the journal, and the lines indicate the degree of collaboration/connection).

Journal co-citation analysis further shows that the *Journal of Controlled Release* (3,870 times), *International Journal of Molecular Sciences* (3,141 times), and *Journal of Extracellular Vesicles* (2,449 times) are highly influential journals in this field’s knowledge base (Figure 6).

**FIGURE 7:**
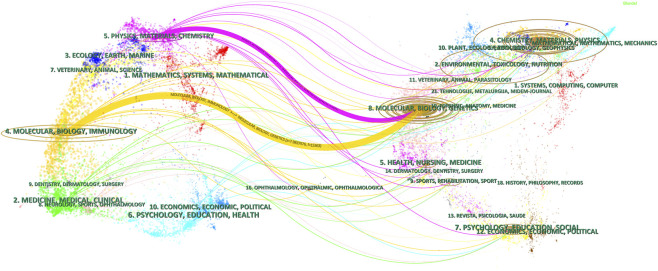
Overlaying dual-map visualization of disciplinary field flows.

The dual-overlay analysis in Figure 7 indicates that this field is simultaneously supported by both material science and molecular biomedical research. The path from Physics, Materials, Chemistry to Molecular, Biology, Genetics highlights the significance of biomaterials and nanotechnology in the construction of exosome drug delivery systems; the path from Molecular, Biology, Immunology to Molecular, Biology, Genetics reflects the importance of biological mechanisms such as exosome transport, immune compatibility, and BBB penetration efficiency. This suggests that exosome-mediated BBB drug delivery is an interdisciplinary research direction connecting nanomedicine, molecular pharmacology, and CNS disease therapy.

### 3.6 Citation analysis

**FIGURE 8:**
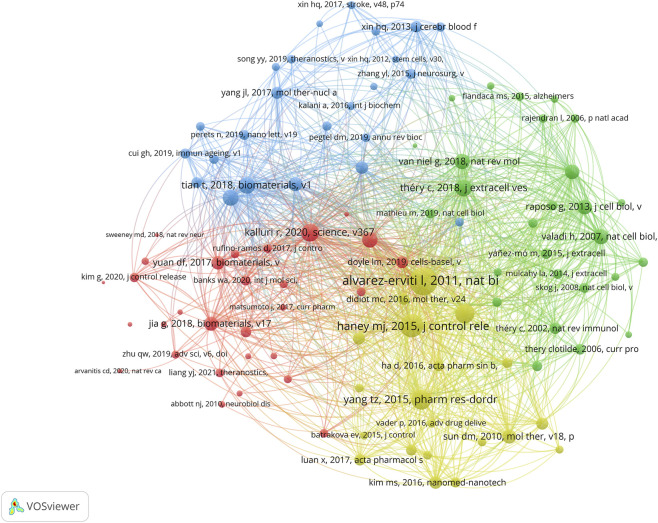
Network visualization map of literature co-citation analysis (node size represents the total connection strength of the literature, and the lines indicate the degree of collaboration/connection).

Co-citation analysis of the literature can illustrate the overall distribution of foundational publications in this research field. As shown in Figure 8, among the core publications with high total link strength, the study by Alvarez-Erviti L published in *Nature Biotechnology* in 2011 (total link strength 4443, cited 381 times) ranks first, followed by the study by Haney MJ published in *Journal of Controlled Release* in 2015 (total link strength 3382, cited 243 times) and the study by Yang TZ published in *Pharmaceutical Research* in 2015 (total link strength 2771, cited 210 times). These highly co-cited publications mainly focus on targeted exosome delivery to the brain, engineered exosome drug loading, and drug transport across the BBB, indicating that these directions form an important part of the early knowledge base in this field.

**FIGURE 9:**
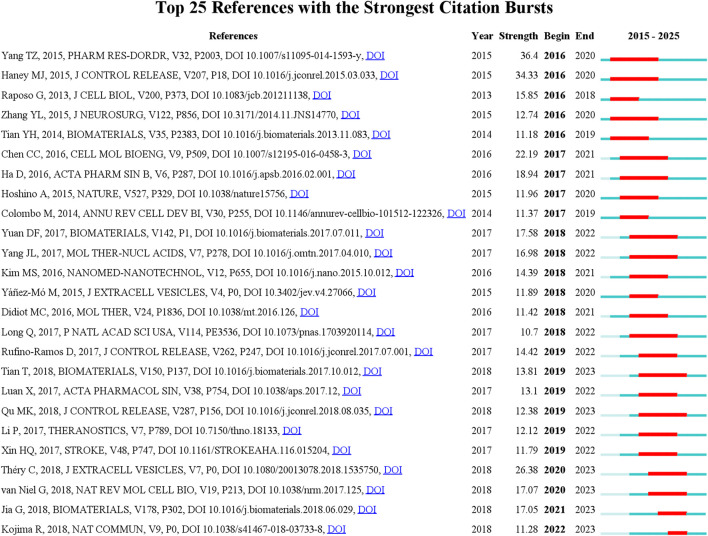
References outbreak. Note: The red section indicates the period during which the article was heavily cited.

Citation bursts show that the study by Yang TZ, published in 2015, had the strongest burst intensity between 2016 and 2020, followed by the studies by Haney MJ, published in 2015, and Théry C, published in 2018 (Figure 9). These burst publications mainly focus on exosome drug delivery across the BBB, CNS disease delivery applications, and EV characterisation standardisation, indicating that early research hotspots focused on validating drug-delivery feasibility and establishing methodological standards.

### 3.7 Keyword analysis

**FIGURE 10:**
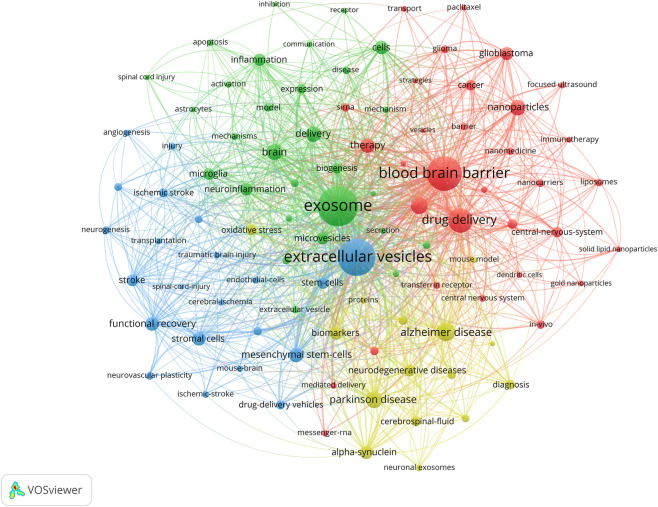
Keyword co-occurrence map (node size represents the total connection strength of the author, and the lines indicate the degree of collaboration/connection, WoSCC).

**FIGURE 11:**
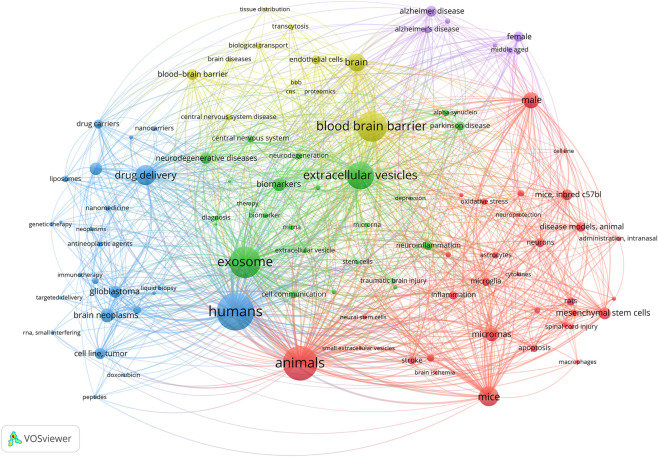
Keyword co-occurrence map (PubMed).

Keyword analysis revealed common themes and differences between the two databases. In WoSCC, the keywords with the highest total link strength included “exosome”, “extracellular vesicles”, “blood–brain barrier”, “drug delivery” and “Alzheimer’s disease”; in PubMed, the high-frequency terms included “human”, “animal”, “exosome”, “blood–brain barrier” and “extracellular vesicles”, which is related to PubMed’s greater emphasis on biomedical subject indexing (Figures 10, 11).

**FIGURE 12:**
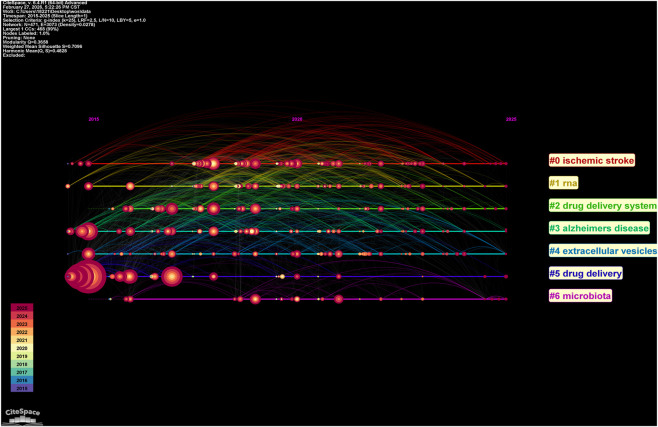
Keyword clustering evolution map (WoSCC).

**FIGURE 13:**
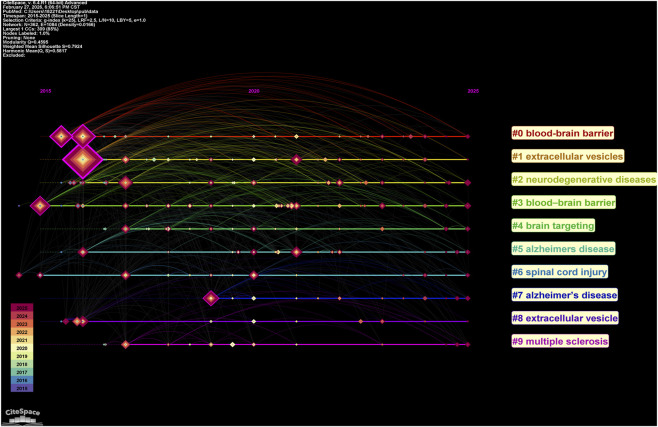
Keyword clustering evolution map (PubMed).

The keyword cluster evolution results in Figures 12, 13 show that the field is gradually shifting from basic molecular research towards drug delivery system design and disease applications. In WoSCC, Cluster #1 RNA and Cluster #2 drug delivery system reflect the significant association between basic microscopic molecular research and carrier design as well as drug delivery; Cluster #6 microbiota increased in activity after 2018, suggesting that gut–brain axis-related mechanisms began to receive more attention. In PubMed, cluster labels such as #3 blood-brain barrier, #6 traumatic brain injury, and #10 spinal cord injury indicate a greater focus on anatomical barriers, disease models, and translational applications. Overall, both databases established a research framework in which extracellular vesicles serve as carriers, with neurodegenerative diseases such as Alzheimer’s disease as the primary therapeutic targets.

**FIGURE 14:**
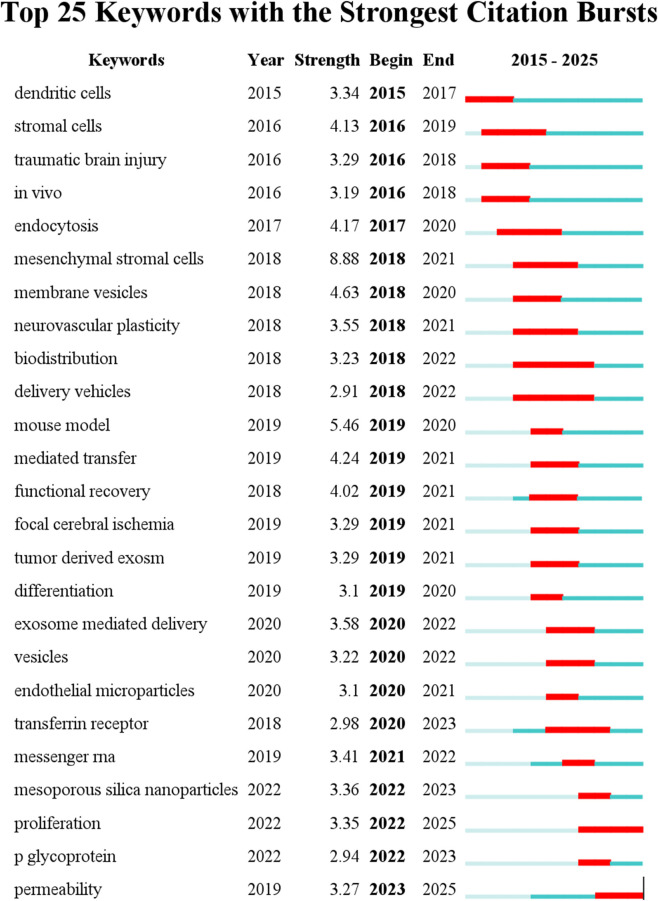
Keywords outbreak. Note: The red part indicates that this keyword was the main research focus during this period.

**FIGURE 15:**
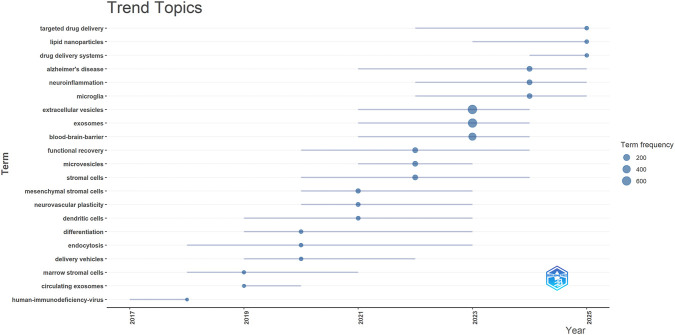
Research Topic Evolution Trend Diagram. Note: When the ending is highlighted in blue, it indicates that the topic represented by the term will become a key research direction in the future.

The keyword burst analysis further reflects the shift in research focus. “Mesenchymal stromal cells” and “mouse model” experienced bursts from 2018 to 2021 and 2019–2020, respectively, indicating that at that time, the clinical applications of exosomes were still at the stage of animal experiments in basic research. In recent years, keywords such as “permeability” and “proliferation” suggest that researchers are paying more attention to the penetration effect of exosome-mediated drugs across the blood–brain barrier (Figure 14). The thematic trend map also shows that “drug delivery”, “nanoparticles”, “blood-brain barrier” and “extracellular vesicles” have emerged as current hotspots, further supporting the view that the field is shifting from conceptual research on exosomes to operationally engineered drug delivery strategies (Figure 15).

### 3.8 Multi-database stability verification

To further verify the stability of the bibliometric analysis results, this study introduced Embase as a supplementary validation database, based on a comparative analysis between WoSCC and PubMed, and compared it with WoSCC.

Pearson correlation analysis showed that, in terms of annual publication trends, WoSCC exhibited high consistency with the two supplementary databases. Specifically, the Pearson correlation coefficient between WoSCC and PubMed was 0.985 (p < 0.001), and between WoSCC and Embase was 0.982 (p < 0.001). These results indicate that the growth trend of publications in the WoSCC database in this field has been validated in both PubMed and Embase (Table 3).

**TABLE 3:** Pearson correlation analysis of annual publication trends in different databases.

Comparison	Year range	Pearson r	Sig. (2-tailed)
WoSCC vs. PubMed	2015–2025	0.985	<0.001
WoSCC vs. embase	2015–2025	0.982	<0.001

The consistency of the Top 10 keywords, countries/regions, and journal sources was analysed using the Jaccard similarity coefficient and the Spearman correlation coefficient. The stability of the main country/region distribution was relatively high, with Jaccard coefficients of 0.818 and 0.667 for WoSCC versus PubMed and WoSCC versus Embase, respectively, and Spearman rho values of 0.983 (p < 0.001) and 0.762 (p = 0.028), respectively. For journal sources, the consistency between WoSCC and PubMed was high (Jaccard = 0.818, Spearman rho = 0.933, p < 0.001); the overlap between WoSCC and Embase was lower, but the ranking correlation was good (Jaccard = 0.429, Spearman rho = 0.886, p = 0.019). For keywords, the Jaccard coefficient between WoSCC and PubMed was 0.462, with a Spearman rho of 0.886 (p = 0.019); between WoSCC and Embase, the Jaccard coefficient was 0.429, with a Spearman rho of 1.000 (p < 0.001). These results indicate that supplementary validation of WoSCC analysis results using PubMed and Embase shows good stability at the macro level, while the overlap in keywords with Embase journal sources is relatively low, which may be related to differences in subject term indexing systems across databases (Table 4).

**TABLE 4:** Jaccard coefficients and Spearman correlation analysis of the Top 10 bibliometric indicators.

Validation item	Comparison	Jaccard coefficient	Shared items	Spearman rho	Sig. (2-tailed)
Country/region	WoSCC vs. PubMed	0.667	9	0.983	<0.001
Country/region	WoSCC vs. embase	0.818	8	0.762	0.028
Journal	WoSCC vs. PubMed	0.818	9	0.933	<0.001
Journal	WoSCC vs. embase	0.429	6	0.886	0.019
Keyword	WoSCC vs. PubMed	0.462	6	0.886	0.019
Keyword	WoSCC vs. embase	0.429	6	1.000	<0.001

## 4 Discussion

### 4.1 General information

Bibliometric results show that from 2015 to 2025, research on exosome-mediated drug delivery across the BBB has steadily expanded, with two significant peaks in 2019–2020 and 2024–2025. Analysis of highly cited literature indicates that the first growth peak was driven by concentrated efforts to address the inherent limitations of exosome carriers. During this period, research focused on addressing deficiencies in exosome storage stability, yield, purity, and targeting, which led to extensive exploration of optimized administration routes, improved drug-loading methods, and surface modification techniques (Ha et al., 2016). The second growth peak may be associated with the deep integration of nanotechnology. As research has extended into the field of biomimetic nanovesicles, using nanotechnology to enhance drug capability across the BBB, increase targeted accumulation in the brain, and reduce systemic side effects has become a mainstream direction in recent years (Huang et al., 2024a). The evolution of research in these two stages outlines the transition of exosome-based brain drug delivery from fundamental property improvement to the development of high-performance biomimetic systems.

From the perspective of countries, institutional distribution, and their collaboration networks, a total of 59 countries and 1,417 academic institutions worldwide have participated in research in this field. Among them, China and the United States are the primary contributors, jointly producing 795 publications and enhancing their research influence through frequent international collaborations (such as 52 collaborations between China and the United States). Harvard University in the United States and the Chinese Academy of Sciences are the major contributing institutions. Additionally, among the top ten institutions by publication volume in this field, three are from the United States, and six are from China, indicating that research teams from both countries are leading the field’s development. At the same time, the distribution differences of the top ten institutions in the publication volume between the two databases reveal the deeper logic of the ‘medical-engineering integration’ in the field of exosome-mediated drug delivery across the blood-brain barrier: the WoSCC database, as a multidisciplinary database, includes a large number of journals in materials science, chemistry, and engineering, and early exosome research involved substantial engineering modifications and material characterization of nanocarriers, which are strengths of these comprehensive universities and technical institutions on the list; in contrast, the PubMed database is strictly focused on the biomedical field, with the top chart list consisting mostly of medical schools with clinical backgrounds, indicating that the research focus has shifted from carrier construction to preclinical pharmacological validation and evaluation of therapeutic effectiveness (AlRyalat et al., 2019).

In terms of journals and authors publishing articles, the global benchmark top journal in the field of drug delivery and controlled release formulations, the *Journal of Controlled Release*, has published the most relevant articles. At the same time, *ACS Nano*, widely recognized as a leading comprehensive journal in the global field of nanoscience and technology, has also published several influential studies that drive the development of nanobiomedicine worldwide. Zhang Y is one of the more active and representative authors in this field. The top three most-cited papers show that most studies mainly focus on exosome-based drug delivery systems, using endogenous nanovesicles to deliver drugs to the brain to prevent and treat a range of brain diseases, including brain cancer and neurodegenerative disorders (Haney et al., 2015; Yang et al., 2015). For example, Alvarez-Erviti L and others intravenously injected RVG-targeted exosomes into wild-type mice to deliver siRNA specifically to neurons, microglia, and oligodendrocytes in the brain (Alvarez-Erviti et al., 2011). This strongly knocked down the mRNA and protein expression of the Alzheimer’s disease treatment target BACE1, highlighting the therapeutic effect of exosome-mediated drug delivery.

From the analysis of keyword emergence and thematic evolution, research in this field from 2015 to 2025 can be divided into three stages. The early stage (2015–2018) primarily focused on selecting exosome sources and their basic transport mechanisms. The high emergence intensity of “dendritic cells” and “mesenchymal stromal cells” reflects early efforts by researchers to identify donor cells with good biocompatibility and high yield (Phinney and Pittenger, 2017); meanwhile, the appearance of “endocytosis” indicates that this period concentrated on elucidating the basic pathways by which exosomes cross the endothelial cell layer (Mulcahy et al., 2014). The mid-stage (2019–2022) shifted focus to enhancing the targeting efficiency of exosomes. Due to the limited natural targeting of exosomes, researchers began using brain highly expressed receptors, such as the “transferrin receptor,” and modified ligands (such as the T7 peptide) on the vector surface through genetic engineering to improve receptor-mediated endocytosis efficiency (Johnsen et al., 2014). The emergence of “ischemic stroke” and “functional recovery” during this stage indicates that research goals had extended from mere brain delivery to evaluations of therapeutic outcomes such as neural function repair (Xin et al., 2013). In the recent stage (2023–2025), “targeted drug delivery”, “lipid nanoparticles”, and “neuroinflammation” have become core terms. This indicates that the research focus has shifted from simply crossing the BBB to the precise regulation of the brain microenvironment, including microglia-mediated neuroinflammation.

### 4.2 Potential exploration of hotspot mechanisms

The increase in publications in this field cannot, in itself, explain why certain topics become research hotspots. Therefore, this section will discuss the underlying mechanisms behind the main research hotspots, especially BBB penetration, efflux transporter evasion, and neuroinflammation-related targeting.

#### 4.2.1 Molecular mechanisms of receptor-mediated transcytosis

Unlike traditional nanoparticles, which mainly rely on inefficient paracellular diffusion, the core mechanism by which exosomes cross the BBB lies in receptor-mediated transcytosis. Specific receptors, such as the transferrin receptor (TfR), insulin receptor, low-density lipoprotein receptor-related protein 1 (LRP1), and nicotinic acetylcholine receptor, are highly expressed on the surface of brain microvascular endothelial cells. By modifying exosome surfaces with corresponding ligands (such as the RVG peptide, T7 peptide, or Angiopep-2), the endocytic program of endothelial cells toward exosomes can be effectively initiated (Alvarez-Erviti et al., 2011; Abbott et al., 2010). At the pharmacokinetic level, the density of ligand modification is not linearly and positively correlated with brain entry efficiency. Excessively high ligand concentrations may increase exosomes’ affinity for receptors, leading to their retention within cells and routing to the endosome-lysosome pathway for degradation. Studies have shown that a moderate ligand density, in combination with the naturally carried tetraspanins of exosomes, can trigger clathrin-mediated endocytosis, thereby helping exosomes evade lysosomal degradation and ultimately be released into the brain parenchyma via basal exocytosis from endothelial cells (Mulcahy et al., 2014; Johnsen et al., 2014). This receptor-ligand interaction-based transport strategy significantly enhances the targeting efficiency of exosomes to the central nervous system, thereby improving the bioavailability of the associated drugs.

#### 4.2.2 Pharmacokinetic mechanisms for circumventing efflux transporters and reversing multidrug resistance

In addition to the physical barrier, the BBB also possesses a biochemical barrier composed of high-efflux transporters. Among them, ATP-binding cassette (ABC) transporters, represented by P-gp and BCRP, can recognize and efflux lipophilic drugs that have entered brain microvascular endothelial cells. This is the main mechanism underlying CNS drug delivery failure and the emergence of multidrug resistance (MDR) (Tournier and Langer, 2025; Chai et al., 2022). As drug-delivery carriers, exosomes offer advantages in pharmacokinetics by circumventing efflux mechanisms. Firstly, exosomes enter cells via receptor-mediated endocytosis, and their endogenous lipid bilayer physically encapsulates drug molecules within the lumen, effectively preventing them from contacting the binding sites of transport substrates such as P-gp on the cell membrane, thereby enabling carrier-mediated delivery that evades recognition by efflux pumps (Abbott et al., 2010; Batrakova and Kim, 2015). Secondly, exosomes can bypass lysosomal degradation via specific intracellular transport pathways and release drugs directly into the cytoplasm or nucleus of target cells via membrane fusion, thereby significantly enhancing the distribution volume and bioavailability of drugs in the brain parenchyma (Chai et al., 2022; Batrakova and Kim, 2015).

#### 4.2.3 Targeting mechanisms of microglial polarisation and neuroinflammation

In the context of intervention strategies for neurodegenerative diseases, the limitations of targeting neurons alone have drawn widespread attention. As the resident immune cells of the CNS, microglia exhibit phenotypic polarization that plays a bidirectional regulatory role in disease progression: under pathological conditions, microglia predominantly polarize to the M1 pro-inflammatory type, secreting large amounts of pro-inflammatory factors; during the tissue repair phase, they tend to shift to the M2 anti-inflammatory type, functioning to clear amyloid protein deposits and secrete neurotrophic factors (Long et al., 2020). Studies have shown that exosomes exhibit an innate tropism towards microglia. Utilizing this characteristic, researchers have loaded specific microRNAs (such as miR-124) or small-molecule drugs (such as curcumin) into exosomes, enabling them to cross the blood-brain barrier (BBB) and enter the brain parenchyma. Subsequently, activated M1-type microglia phagocytose drug-loaded exosomes, thereby inhibiting pro-inflammatory signaling pathways such as TLR4/NF-κB and inducing a phenotypic shift from pro-inflammatory M1 to neuroprotective M2 (Ke et al., 2026; Li et al., 2023). This immune modulation strategy targeting the core driving factors of the disease microenvironment provides an important direction for achieving systemic intervention in neurodegenerative diseases.

### 4.3 Evolution of exosomes from different sources in BBB delivery and current status of clinical translation

The selection of exosome sources has gradually become an important issue in the study of BBB drug delivery mechanisms. Exosomes from different sources may affect certain aspects of mechanism research due to differences in their surface molecules, immune responses, and interactions with the BBB. Therefore, this section will further discuss the evolution of exosomes from different sources in BBB delivery and their current status in clinical translation.

Based on retrieval results from the WoSCC and PubMed databases in this study, research from 2015 to 2025 shows that mammalian cell-derived exosomes have dominated BBB delivery, with gradual expansion toward plant-derived vesicles. Mammalian cell-derived exosomes, especially those from MSCs, immune cells, and tumor cells, are the most common. MSC-derived exosomes have low immunogenicity, good biocompatibility, and high nucleic acid and drug-loading potential. They have thus played a dominant role in BBB-targeted drug delivery for CNS diseases (Rehman et al., 2023; Wang et al., 2022a). Immune cell-derived exosomes, due to their tropism for brain lesions, often show better targeting within the cerebral lesion microenvironment. However, their performance depends on inflammatory polarization, and issues with drug-delivery stability and reproducibility limit translation (Wang et al., 2021). Tumor cell-derived exosomes offer unique advantages in BBB penetration and homotypic targeting, but their tumor-promoting, metastasis-promoting, and immunosuppressive risks warrant caution in clinical translation (Morad et al., 2019). Plant-derived exosomes were initially reported less frequently, but since 2021, studies have increased, with this trend becoming more pronounced between 2023 and 2025. This suggests that plant-derived exosomes are considered an alternative platform, particularly for their raw material acquisition, cost control, scalable production, and potential for non-invasive delivery, such as nasal-to-brain strategies (Niu et al., 2021; Kim et al., 2023; Jin et al., 2025). However, based on the existing overall literature, plant-derived exosomes should be regarded more as a rapidly emerging new direction, while the ones that truly dominate are still MSCs and other engineered mammalian-derived exosomes (Rehman et al., 2023; Niu et al., 2021; Kim et al., 2023). In other words, the evolution of drug-delivery exosomes is not about simply substituting sources but involves differentiating based on trade-offs among BBB penetration, immunogenicity, scalability, drug loading, and regulatory acceptability.

From the perspective of current clinical translation, exosome-mediated drug-targeted therapy for CNS diseases is still primarily at the preclinical and early clinical exploration stages. As summarized in Table 5, the literature included in this study and publicly registered information indicate that current research has covered AD, PD, ischemic stroke, spinal cord injury, amyotrophic lateral sclerosis, glioma, and depression/anxiety-related disorders. Among them, some studies, such as NCT04202770, focusing on focused ultrasound-assisted exosome delivery for treatment-resistant depression, anxiety, and dementia, and NCT04388982 related to Alzheimer’s disease, have entered the registration stage on ClinicalTrials.gov. (ClinicalTrials.gov., 2019; ClinicalTrials.gov., 2020). However, the U.S. Food and Drug Administration (FDA) has explicitly stated that no exosome products have been approved to date (Administration, 2019). The above situation once again illustrates that, although this field has considerable translational potential, it is still at an early stage of transitioning from experimental research to clinical validation. The heterogeneity of exosome sources, product standardization, long-term safety evaluation, and scalable production pathways remain immature and are key issues for further clinical advancement (Rehman et al., 2023; Wang et al., 2022a).

**TABLE 5:** Summary of studies on targeted drug delivery for CNS diseases based on exosomes.

Diseases	Source category	Main source	Route of administration	BBB involvement	Current stage
Alzheimer’s disease Sun et al. (2025b)	Mammalian cell-derived	Engineered stem cell-derived exosomes (hUC-MSC-related platform)	Intranasal administration	Yes (nose-to-brain delivery)	Exploratory/preclinical
Alzheimer’s disease Mi et al. (2025)	Plant/fungus-derived	Ganoderma lucidum-derived exosome-like nanovesicles	Intranasal administration	Yes (nose-to-brain delivery)	Exploratory/preclinical
Parkinson’s disease Huang et al. (2024b)	Mammalian cell-derived	Umbilical cord MSC-derived exosomes	Intranasal administration	Yes (nose-to-brain delivery)	Exploratory/preclinical
Parkinson’s disease Mondal et al. (2025)	Mammalian cell-derived	Dental pulp stem cell-derived sEVs	Intranasal administration	Yes (nose-to-brain delivery)	Exploratory/preclinical
Parkinson’s disease Sul et al. (2024)	Mammalian cell-derived	Adipose-derived stem cell EVs	Systemic administration	Yes	Exploratory/preclinical
Glioma/Glioblastoma Niu et al. (2021)	Plant-derived	Grapefruit-derived EVs	Systemic administration	Yes	Exploratory/preclinical
Glioma/Glioblastoma Wang et al. (2021)	Mammalian immune cell-derived	Neutrophil-derived exosomes	Intravenous injection	Yes	Exploratory/preclinical
Glioma/Glioblastoma Cui et al. (2023)	Mammalian cell-derived	Serum/endogenous exosomes coating nanomicelles	Systemic administration	Yes	Exploratory/preclinical
Glioma/Glioblastoma Kim et al. (2023)	Plant-derived	Ginseng-derived exosome-like nanoparticles	Systemic administration	Yes	Exploratory/preclinical
Glioma/Glioblastoma Chu et al. (2025)	Mammalian tumor cell-derived	Tumor-derived exosomes	Systemic administration	Yes	Exploratory/preclinical
Ischemic stroke Rohden et al. (2021)	Mammalian cell-derived	Adipose MSC-derived EVs	Intranasal administration	Yes (nose-to-brain delivery)	Exploratory/preclinical
Ischemic stroke Dave et al. (2023)	Mammalian cell-derived	Brain endothelial cell-derived EVs	Intravenous administration	Yes	Exploratory/preclinical
Ischemic stroke Sun et al. (2025c)	Other EV source	*Lactobacillus* reuteri-derived EVs	Systemic administration	Yes	Exploratory/preclinical
Spinal cord injury Pan et al. (2021)	Mammalian cell-derived	Schwann cell-derived exosomes	Tail vein injection	No(BSCB-related)	Exploratory/preclinical
Spinal cord injury Kong et al. (2025)	Mammalian immune cell-derived	Engineered M2 microglia-derived EVs	Systemic administration	Yes(Angiopep-2-mediated BBB or BSCB targeting)	Exploratory/preclinical
Depression Yu et al. (2022)	Mammalian cell-derived	Engineered RVG-circDYM EVs (HEK293T-related platform)	Systemic administration	Yes	Exploratory/preclinical
Depression Hu et al. (2023)	Mammalian exosome-coated platform	Exosome-sheathed nanogel	Intranasal administration	Yes (nose-to-brain delivery)	Exploratory/preclinical
Depression/anxiety-like behaviors Jin et al. (2025)	Plant/algal-derived	Chlorella vulgaris-derived EVs	Intranasal administration	Yes (nose-to-brain delivery)	Exploratory/preclinical

1. This table only includes original English research articles retrieved from WoSCC and PubMed in this study; reviews, bibliometric analyses, commentaries, and studies solely on biomarkers are not included in the main table. 2. The “stage” in the table is based on information available in the literature itself; studies without clinical registration or formal clinical results are categorized as “exploratory/preclinical”.

### 4.4 Future research directions and feasible strategies

Based on keyword emergence, thematic trend evolution, and cluster evolution diagrams, the future development of this field is not merely a simple expansion of drug-delivering exosome sources but also requires exploring feasible strategies to enhance BBB penetration efficiency, targeted drug delivery, and translational validation capabilities. Therefore, this section focuses on three future directions closely related to bibliometric findings and translational needs.

#### 4.4.1 Design and validation of an AI-Assisted exosome-based drug delivery system across the BBB

AI-assisted design is a promising tool for future studies on exosome-based drug delivery across the BBB, but its true value should not be seen as a ready-made solution that replaces basic experimental validation. A more feasible approach is to use AI for preliminary target screening of candidate exosome delivery systems, optimisation of surface modification design, and prediction of dosage regimens and *in vivo* distribution (Gao et al., 2024). In traditional pharmacological research, screening for exosome surface modification peptides with high BBB permeability often relies on lengthy trial-and-error experiments. However, with the leapfrog development of machine learning and AI molecular modelling technologies, the application of AI will drive the research paradigm from experience-driven to a big-data-based “predict-validate” cycle (Gao et al., 2024; Vora et al., 2023). Specifically, researchers can use AI through deep learning algorithms to integrate multi-source data, including genomic, proteomic, transcriptomic, and single-cell sequencing data from public databases, analyse the dynamic expression patterns of receptors on BBB endothelial cells under different CNS disease states, identify new highly expressed specific membrane protein targets, and intelligently match donor cell sources with natural affinity (Vora et al., 2023; Zhang et al., 2026). Additionally, AI combined with graph neural networks can simulate the complex protein interaction network on the exosome surface, predicting the effects of different modification strategies on carrier stability and immunogenicity, achieving intelligent optimisation of carrier structures (Noury et al., 2025). Nevertheless, the final AI predictions must be combined with a standardised empirical validation system. Therefore, future research could establish a complete “predict-validate-feedback” closed-loop process: first using AI to screen candidate exosome sources and targets and predict the effects of surface modification strategies; next, assessing the transport efficiency of drug-carrying exosomes across the BBB in animal models, evaluating changes in BBB integrity, and using *in vivo* tracing to analyse the dynamic relationships governing drug distribution in peripheral organs, brain retention, and lesion enrichment, thereby verifying the efficacy of candidate exosome drug delivery and modification strategies; finally, feeding the experimental results back into the AI prediction model to progressively improve prediction accuracy (Gao et al., 2024).

It should be noted that AI-assisted exosome design still faces issues such as heterogeneity in training data, inconsistent standards for exosome characterisation, insufficient sample sizes, poor reproducibility of experimental results, and limitations in cross-model applications. At the same time, exosome research itself imposes high requirements on sample handling, isolation and purification, characterisation, and functional validation. If the foundational data includes a significant amount of journal data, AI model predictions are also difficult to reproduce consistently across different experimental systems (Lai et al., 2022). Therefore, future research in this field should not merely focus on advancing AI-enabled exosome drug delivery, but also on establishing verifiable datasets, clear predictive metrics, and reproducible experimental validation protocols.

#### 4.4.2 Construction and validation of biomimetic hybrid nanovesicles

The presence of the BBB is a major obstacle to CNS therapy, whereas biomimetic hybrid nanovesicles offer a new engineering approach for exosome-based drug delivery across the BBB. Natural exosomes have good biocompatibility, low immunogenicity, and the ability to cross the BBB, but also face limitations such as low drug-loading efficiency, significant source heterogeneity, large batch-to-batch variation, and difficulties in quality control (Rehman et al., 2023; Mehdizadeh et al., 2025). Therefore, in recent years, the development of hybrid systems integrating natural and synthetic components has become a major research trend to improve the brain-targeting specificity of exosomes. Increasingly, researchers are combining natural exosomes with synthetic nanocarriers such as liposomes, polymer nanoparticles, and cell membrane-coated nanoparticles to achieve the biocompatibility of natural vesicles and the structural designability of synthetic nanocarriers (Sun et al., 2025a). Specifically, exosome-liposome hybrid vesicles can enhance brain delivery stability and BBB transport efficiency by integrating the natural membrane structure of exosomes with liposomes’ high drug-loading capacity. For example, studies have shown that blood-derived exosome-liposome hybrid nanovesicles can enhance brain penetration and are used for glioma combination therapy research (Wang et al., 2024b). Polymeric nanoparticles (NPs) can effectively deliver gene therapy drugs across the BBB; for instance, Angiopep-2-modified CRISPR-Cas9 nanocapsules have been used for glioblastoma gene therapy and demonstrated potential for BBB crossing and tumour tissue targeting (Zou et al., 2022). Biomimetic nanoparticles (MNPs) derived from cell membranes of autologous red blood cells, white blood cells, and others also exhibit high biocompatibility, significantly reducing their immunogenicity, and their surface-enriched homologous proteins can facilitate BBB penetration and selectively target homologous pathogenic cells for therapeutic drug delivery (Li et al., 2020; Wang et al., 2022b).

However, innovative efforts in the field of bionic hybrid nanovesicles should not only focus on developing new hybrid carriers but also on establishing a reproducible, comparable engineering evaluation system. Future research should incorporate preparation processes, quality standards, storage stability, and regulatory acceptability into the design at an early stage, rather than considering clinical translation only after completing animal experiments (Lai et al., 2022). In summary, only by simultaneously addressing delivery efficacy and quality control can bionic hybrid nanovesicles gradually move from laboratory concepts to clinical applications.

#### 4.4.3 Validation of drug delivery pathways and mechanisms of the gut-brain axis

The gut-brain axis drug delivery strategy provides a novel approach for exosome-mediated transport across the BBB, distinct from traditional intravenous and systemic administration. The gut microbiota and the central nervous system form a bidirectional regulatory network involving endocrine, immune, and neural pathways, offering a new mechanistic perspective and a delivery route for non-invasive exosome-based drug delivery (Margolis et al., 2021). Gut microbiota- or probiotic-derived extracellular vesicles are key mediators of gut-brain axis communication, capable of transporting bioactive substances, such as proteins and nucleic acids (e.g., miRNAs), into the circulatory system and influencing the brain microenvironment by modulating peripheral immunity, neuroinflammation, and metabolic signalling (Uceda et al., 2023). Studies suggest that bacterial exosomes derived from symbiotic gut microbiota may impact the pathological progression of Alzheimer’s disease by regulating microglial inflammatory states and β-amyloid deposition via the gut-brain axis (Xie et al., 2025). Moreover, exosome engineering methods can manipulate the microbiome and regulate the molecular cargo of gut-derived vesicles, endowing them with potential as therapeutic carriers (Lai et al., 2022). In terms of delivery routes, Gong et al., based on the presence of gut-derived macrophages in the CNS lymphoma microenvironment, developed an oral drug delivery platform that is recognisable and phagocytosed by gut macrophages, achieving non-invasive brain-targeted delivery through the gut-circulation-brain lesion pathway (Gong et al., 2025). Plant-derived extracellular vesicles have also attracted attention; for example, maca-derived vesicles have been reported to promote serotonin synthesis by modulating gut-brain axis-related pathways, thereby improving depressive behaviours (Hong et al., 2023).

However, current research on gut–brain axis drug delivery is still at the stage of mechanistic exploration, and there are several key issues that need to be validated, such as whether gut-derived, probiotic, or plant-derived vesicles can stably enter the circulatory system and reach brain tissue; whether their function is to deliver drugs directly across the BBB or to indirectly affect the CNS by modulating the gut microbiota, peripheral immunity, and metabolic signals; and whether these vesicles have stable components, a clear mechanism of action, and controllable preparation processes. Therefore, the key issue for future gut–brain axis drug delivery research is not simply to demonstrate its ‘non-invasive’ advantage, but to establish a clear pharmacokinetic and pharmacodynamic evidence chain. Future studies should differentiate between direct drug-delivery effects and indirect regulatory effects by combining analyses of vesicle tracking, brain distribution, changes in gut microbiota, and immune-inflammatory indicators, thereby determining whether it can be truly translated into an assessable brain-targeted drug-delivery strategy.

### 4.5 Analysis of future transformation challenges

AI-assisted design, the development of novel biomimetic hybrid nanovesicles, and new gut-brain axis drug delivery routes, respectively, represent potential future directions for exosome-based BBB-crossing drug delivery in terms of intelligent design, carrier engineering, and expansion of administration pathways. However, this also signals that current research needs to shift from increasing conceptual novelty vertically to systematic validation, from optimising a single platform to lateral comparison between different delivery strategies, and from short-term preclinical efficacy assessment to the construction of a reproducible, scalable, and clinically translatable evaluation system (Rehman et al., 2023; Terstappen et al., 2021).

Firstly, future research needs to establish more unified evaluation standards. Current studies show considerable differences in exosome sources, engineering modification strategies, administration routes, disease models, and detection methods, resulting in a lack of comparability in BBB penetration ability, brain distribution, lesion accumulation, and therapeutic efficacy (Rehman et al., 2023; Lai et al., 2022). Therefore, future studies should evaluate BBB penetration efficiency, brain region distribution, cellular uptake, drug-loading capacity, immune response, therapeutic outcome, and long-term safety under multi-model, multi-disease conditions with unified detection standards (Rehman et al., 2023; Terstappen et al., 2021; Wang et al., 2021).

Secondly, non-target distribution and potential safety should be included in routine evaluations. Systemically administered exosomes may distribute to peripheral organs, depending on the source cells, the administration route, and the targeting strategy (Wiklander et al., 2015). Therefore, future research should further assess distribution in non-target organs such as the liver and kidneys, safety of repeated administration, immune responses, and long-term *in vivo* retention, avoiding superficial judgments of clinical therapeutic potential based solely on brain accumulation results (Kang et al., 2021).

Lastly, standardised preparation, quality control, and regulatory pathways remain major challenges limiting the clinical translation of exosome-based CNS drug delivery. Exosome structure and function are susceptible to variations in source cell status, isolation and purification methods, and drug-loading approaches. MISEV2023 provides updated recommendations on nomenclature, sample handling, isolation methods, characterisation strategies, and functional studies in EV research, which can serve as references for improving research transparency and reproducibility (Welsh et al., 2024). Meanwhile, clinical translation of EV therapeutics also requires attention to manufacturing processes, quality control, potency testing, risk assessment, and regulatory requirements (Wang et al., 2024a). Therefore, the next phase of research should focus on demonstrating that a particular exosome-based delivery strategy is reproducible, comparable, and clinically translatable.

### 4.6 Advantages and limitations

The main advantage of this study lies in the fact that, unlike previous bibliometric analyses that primarily focused on the overall trends of exosome research in CNS diseases, this paper further concentrates on the specialised area of exosome-mediated drug delivery across the BBB, providing a more targeted analysis of BBB-related delivery strategies, core mechanisms, and translational challenges, thereby offering a reference for understanding the research progress and future development of exosomes in brain-targeted drug delivery. At the same time, this study adopted a multi-database verification and comparative analysis strategy. This complementary approach can more effectively cover key literature in the field than single-database studies, thereby enhancing the reliability of the study’s conclusions and providing researchers with a comprehensive map of the current state and anticipated developments in the field.

This study has the following inherent limitations: First, although PubMed and Embase were introduced to supplement and validate the results from WoSCC, access restrictions and methodological limitations prevented the inclusion of databases such as Scopus and CNKI, which limits the research perspective. Second, this study primarily relies on quantitative indicators such as publication volume and citation frequency. Although these well reflect the popularity of research, they may bias evaluations of an article’s influence. Third, since the study retrieved literature from 2015 to 2025, early foundational research prior to 2015 may have been missed. Given that bibliometric results are time-dependent, future publications may alter these findings; therefore, the results of this study should only be understood as a summary of research patterns during this period. Fourth, the study mainly relied on predefined search strategies and database filtering functions for literature selection, without undertaking systematic manual screening or bias risk assessment, which may have led to some peripheral relevant literature being included or excluded.

Additionally, it should be noted that bibliometric analysis can reveal research hotspots, knowledge structures, and thematic shifts, but it cannot directly determine whether a specific drug delivery strategy is truly effective, nor does it indicate that it is ready for clinical translation. As discussed in Section 4.5, exosome-mediated CNS drug delivery research still faces issues such as unstable cellular uptake efficiency, inconsistent BBB penetration, non-targeted distribution, insufficient reproducibility, challenges in large-scale production, and unclear regulatory pathways. Therefore, the hotspots and trends identified in this study are more suitable as references for understanding research directions rather than as direct evidence of maturity for clinical application.

## 5 Conclusion

This study uses the WoSCC database as the primary bibliometric database, supplemented by PubMed and Embase for verification, to examine publication trends, collaboration networks, core journals, keyword evolution, and research hotspots in exosome-mediated drug delivery across the BBB from 2015 to 2025. The results show that this field has developed rapidly over the past decade, with the research focus gradually shifting from basic exosome characterisation to BBB penetration, engineered drug delivery systems, CNS disease applications, and clinical translation evaluation. Multi-database validation further supports the macro-level stability of the WoSCC analysis results regarding annual publication trends, major countries/regions, core journals, and keyword topics.

Unlike previous bibliometric studies that mainly focused on overall CNS exosome trends, this study focuses specifically on exosome-mediated drug delivery across the BBB, discussing mechanisms, the evolution of exosomes from different sources, and barriers to clinical translation. Current research still predominantly involves mammalian-derived exosomes, while plant-source vesicles, AI-assisted design, biomimetic hybrid nanovesicles, and gut-brain axis delivery strategies are gaining attention, and the field remains primarily at the preclinical validation stage. Future research should shift from simply promoting new drug delivery strategies to systematic comparisons of vesicles and delivery platforms from different sources, with a focus on addressing standardised evaluation, quality control, scalable production, long-term safety, and regulatory pathways.

## Data Availability

Publicly available datasets were analyzed in this study. This data can be found here: The datasets analyzed in this study are publicly available from the following sources without specific accession numbers, as they consist of bibliographic and patent records retrieved via the search queries detailed in the Methods section. Data can be reproduced using the provided search strategies. Web of Science Core Collection (WoSCC): https://www.webofscience.com (Clarivate Analytics repository; access may require institutional subscription). PubMed: https://pubmed.ncbi.nlm.nih.gov (National Library of Medicine repository; free public access).
