# Supplementary material for: Global research trends and hotspots of exosome-mediated drug delivery across the blood-brain barrier: a bibliometric study from 2015 to 2025
Source: Front Pharmacol. 2026 Jun 10;17:1835883. doi: 10.3389/fphar.2026.1835883 (PMC13290968; doi:10.3389/fphar.2026.1835883)
Supplement: Supplementary file 1 [file Supplementaryfile1.docx]

Supplementary Material

**
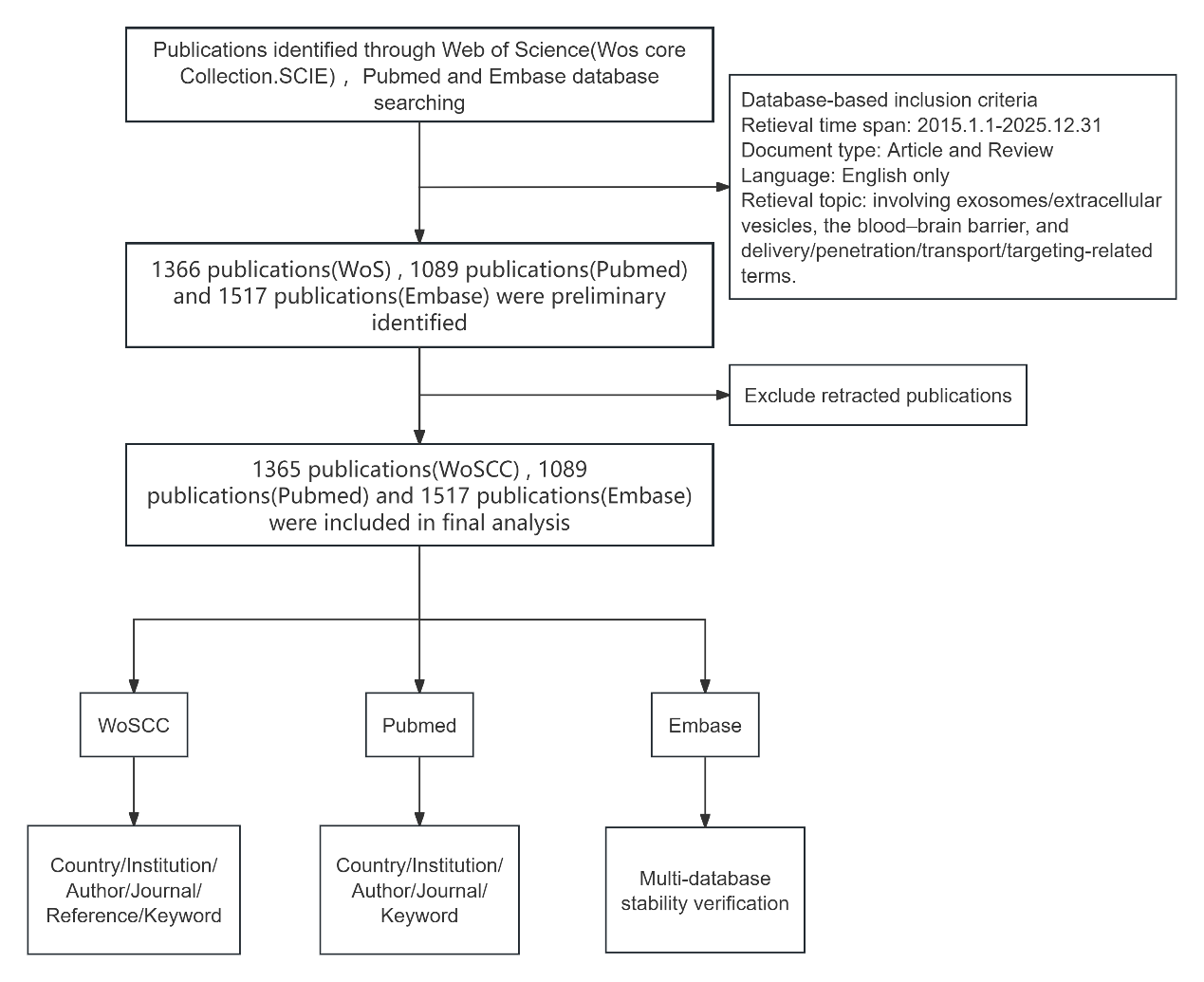
supplementary figure 1.** PRISMA 2020-style flow diagram of database search and filtering process.


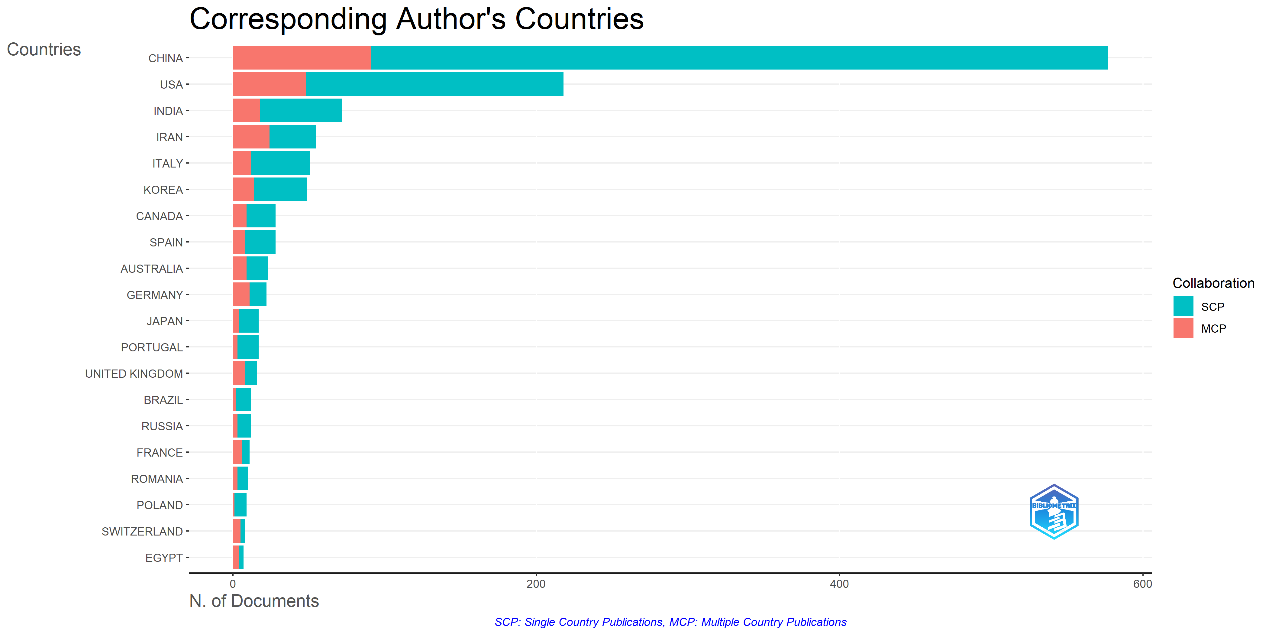
**supplementary figure 2.** Top 20 countries by number of publications(WoSCC).


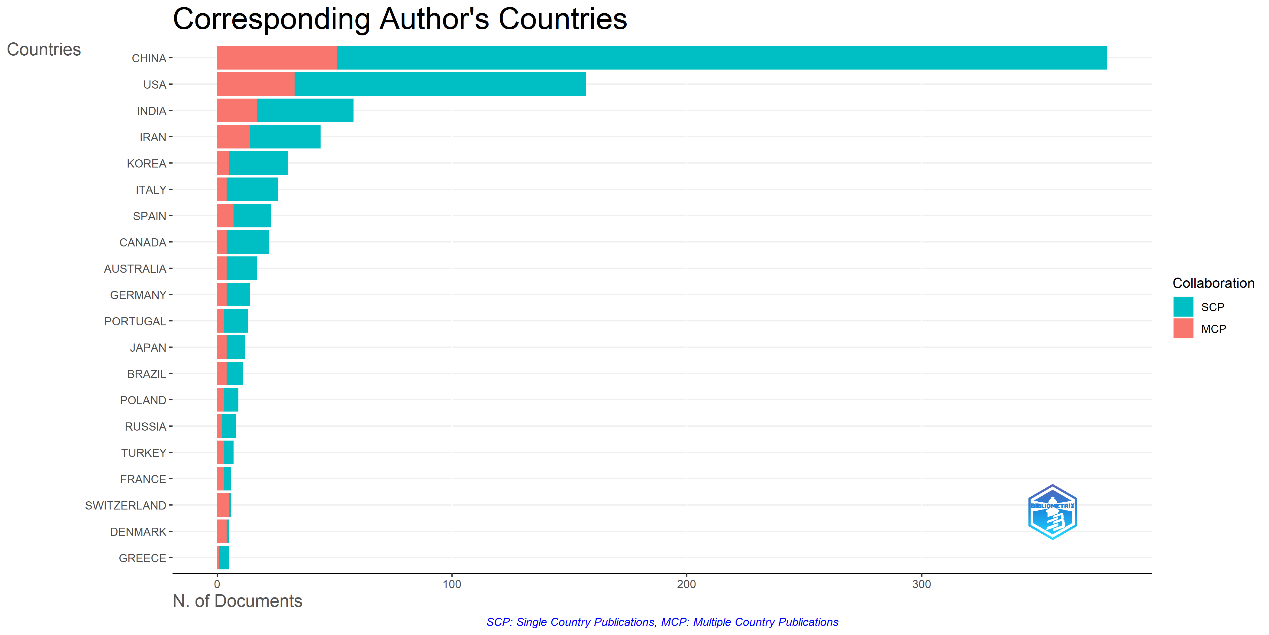
**supplementary figure 3.** Top 20 countries by number of publications(PubMed).


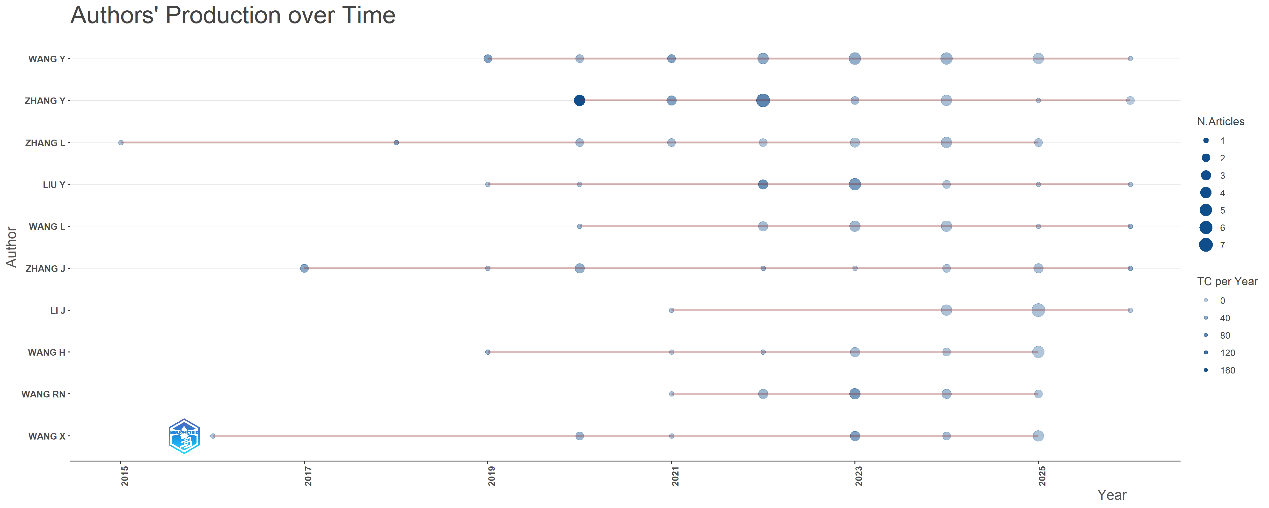
**supplementary figure 4.** Number of publications by the top 10 authors (WoSCC).


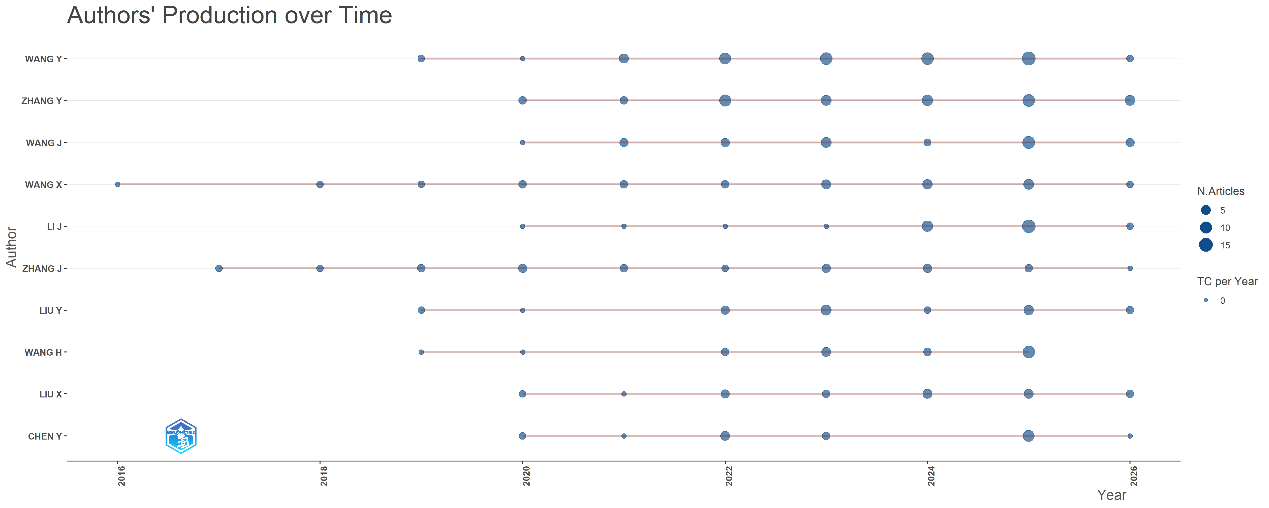
**supplementary figure 5.** Number of publications by the top 10 authors (PubMed).


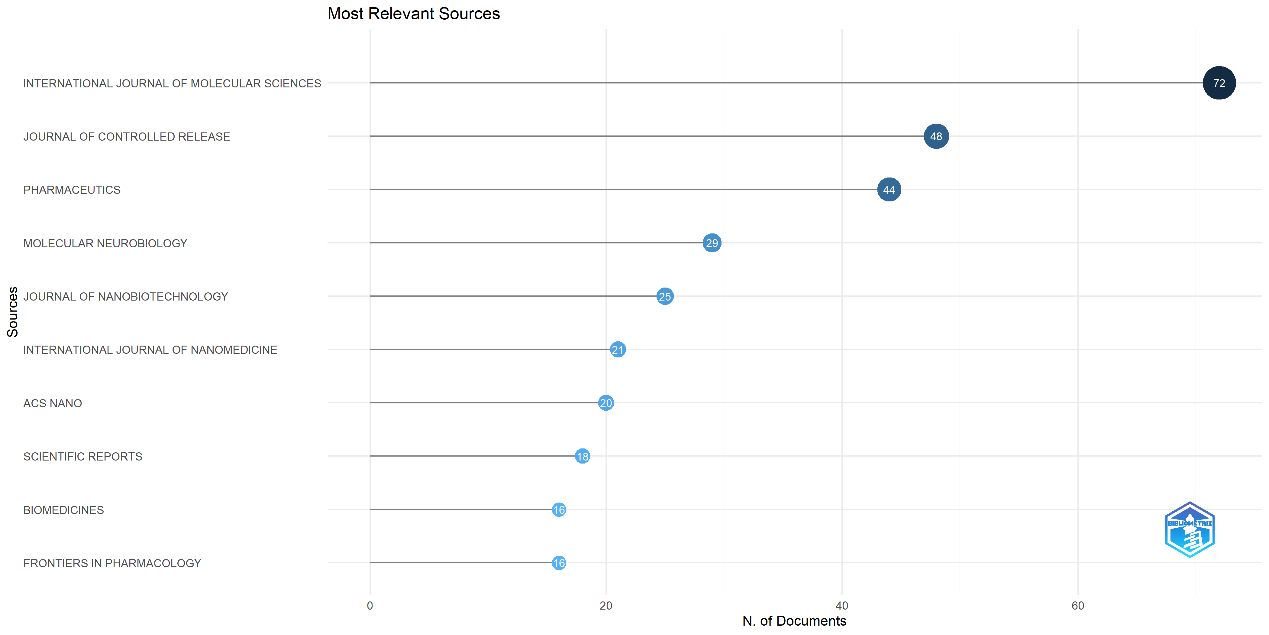
**supplementary figure 6.** Top 10 journals by number of publications(WoSCC).


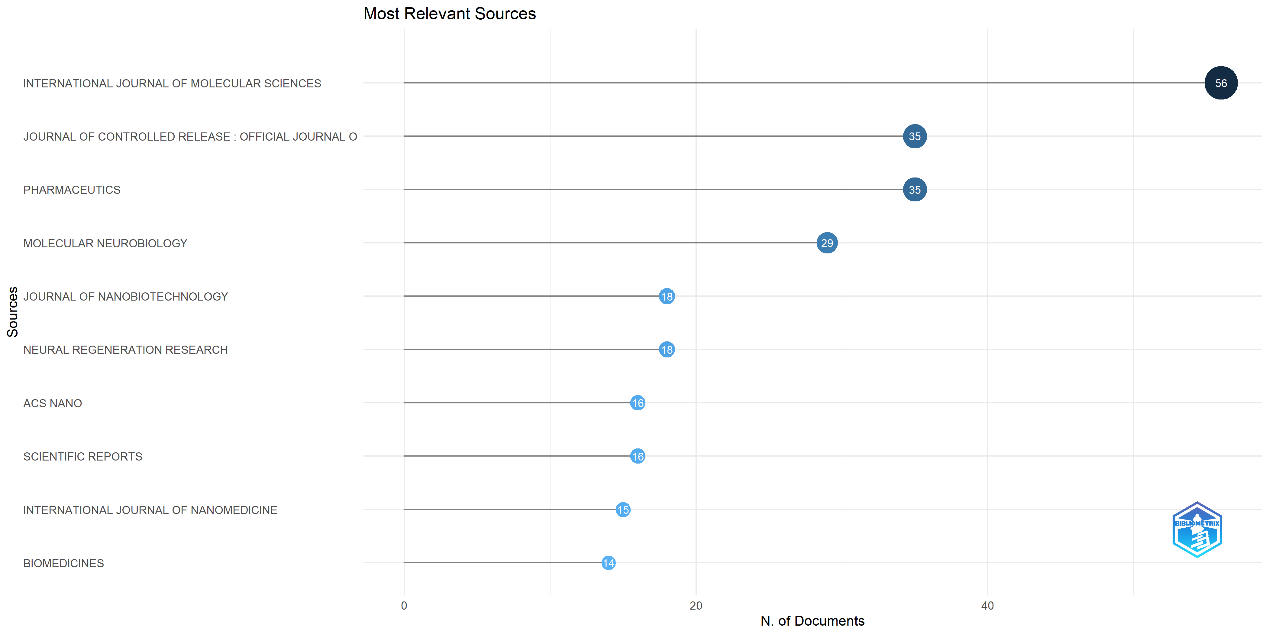
**supplementary figure 7.** Top 10 journals by number of publications(PubMed).
